# Circulating microRNA as promising biomarkers in hypertrophic cardiomyopathy: can advanced cardiac magnetic resonance unlock new insights in research?

**DOI:** 10.3389/ebm.2024.10334

**Published:** 2024-12-18

**Authors:** Olga S. Chumakova, Elena A. Mershina

**Affiliations:** ^1^ National Medical Research Center of Cardiology Named After E. I. Chazov, Moscow, Russia; ^2^ Medical Research and Education Center Lomonosov Moscow State University, Moscow, Russia

**Keywords:** hypertrophic cardiomyopathy, non-coding RNAs, microRNAs, cardiac magnetic resonance, biomarkers, fibrosis, disarray, microvasculature

## Abstract

Hypertrophic cardiomyopathy (HCM) is a genetic cardiac disorder associated with an increased risk of arrhythmias, heart failure, and sudden cardiac death. Current imaging and clinical markers are not fully sufficient in accurate diagnosis and patient risk stratification. Although known cardiac biomarkers in blood are used, they lack specificity for HCM and primarily stratify for death due to heart failure in overt cases. Non-coding RNAs, particularly microRNAs, have emerged as promising biomarkers due to their role in regulating gene expression in both healthy and pathological hearts. Circulating microRNA signatures may dynamically reflect the progression of HCM, offering potential utility in diagnosis and disease monitoring as well as inform biologic pathways for innovative therapeutic strategies. However, studying microRNAs in cardiovascular diseases is still in its early stages and poses many challenges. This review focuses on emerging research perspectives using advanced cardiac magnetic resonance techniques. We presume, that the search for circulating miR signatures associated with specific adverse myocardial features observed on cardiac magnetic resonance imaging - such as fibrosis, disarray, and microvascular disease - represents a promising direction in HCM research.

## Impact statement

We are pleased to submit our manuscript, which highlights a perspective direction in hypertrophic cardiomyopathy research. Our study focuses on non-coding RNAs, specifically microRNAs, as promising cardiac biomarkers, and advanced cardiac magnetic resonance (CMR) imaging as a research tool that could facilitate the discovery of novel circulating miR biomarkers. The current biomarkers are not specific for HCM, and their use is limited by risk stratification for heart failure death. By reviewing recent literature, we discuss the potential to identify specific circulating microRNA signatures linked to adverse microanatomical features of HCM observed using advanced CMR. We aim to engage the HCM scientific community in future interdisciplinary collaborations. The brief review of evolving modalities already applied in some areas of clinical practice may be of interest to a broad audience of practitioners, including cardiologists, radiologists, laboratory specialists, and genetics.

## Introduction

Hypertrophic cardiomyopathy (HCM) is the most common genetic cardiac disorder, characterized by left ventricular (LV) hypertrophy in the absence of abnormal loading conditions [1]. In addition to cardiomyocyte hypertrophy, histological hallmarks include myocardial fibrosis (focal and diffuse), extensive disarray, and microvascular disease (MVD), all linked to arrhythmias, sudden cardiac death (SCD), and heart failure [2–6]. HCM exhibits significant heterogeneity in LV morphology [7], clinical course [8], and genetic etiology, involving both monogenic and polygenic components. Over recent decades, genetic studies have focused on protein-coding genes, identifying sarcomeric gene variants as the primary cause in approximately half of HCM patients [9–11]. This has established sarcomere dysfunction as a crucial mechanism in HCM [12], prompting the development of new treatments, such as cardiac myosin inhibitors [13]. Despite notable advancements, challenges remain in altering disease progression [14]. In clinical practice, there is a need for disease-specific plasma biomarkers to differentiate HCM from secondary LV hypertrophy, enhance risk stratification, and monitor phenotype evolution in preclinical mutation carriers. A fundamental question is how to identify the signaling pathways and regulatory networks that mediate the phenotypic expression of HCM’s complex genetics.

**FIGURE 1:**
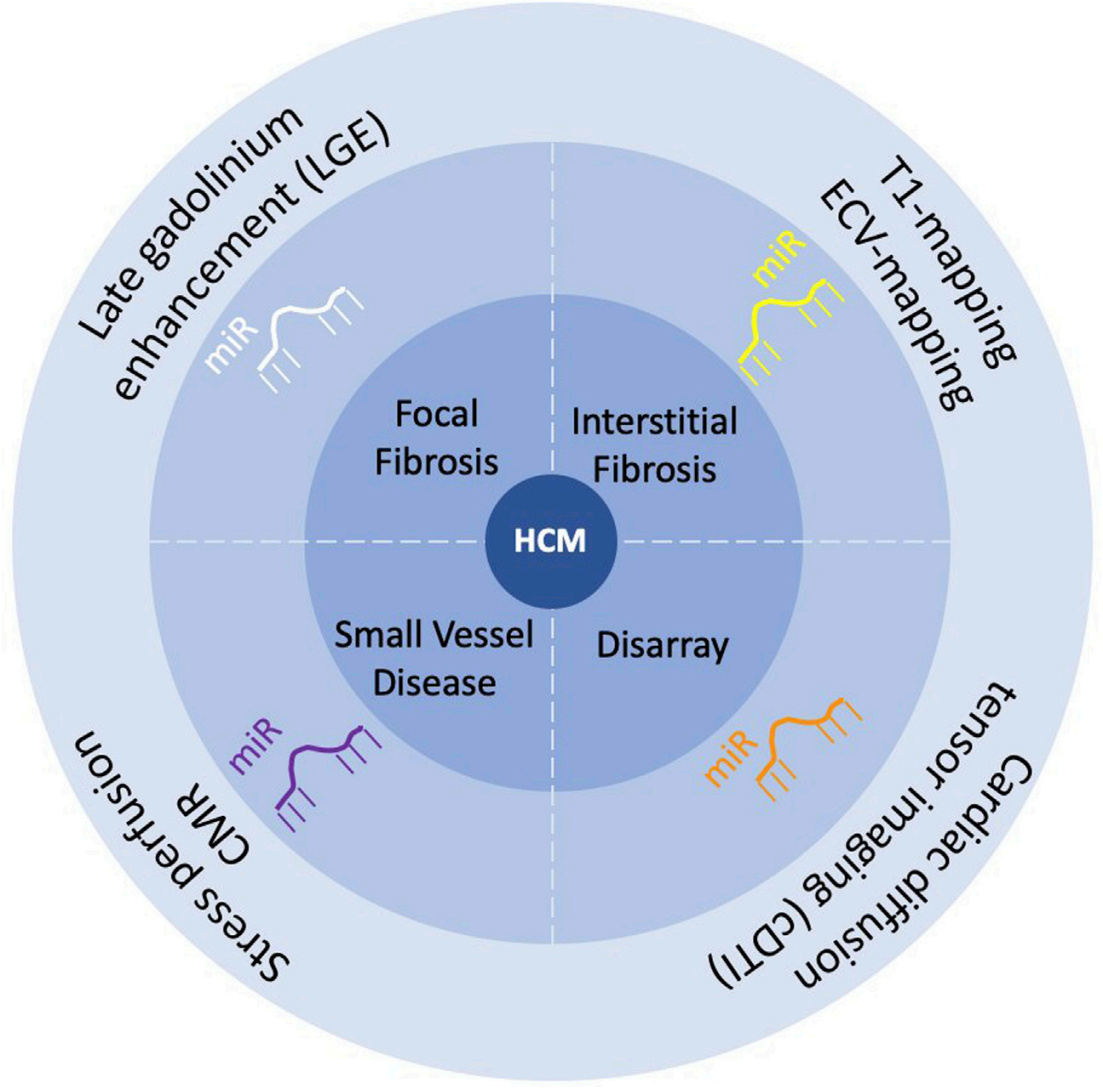
Scheme of the review “Circulating microRNA as promising biomarkers in hypertrophic cardiomyopathy: Can advanced cardiac magnetic resonance unlock new insights in research?”.

This paper reviews micro non-coding ribonucleic acids (microRNAs or miRs), known as negative controllers of gene expression, as promising biomarkers for HCM. A special focus is placed on a) myocardial microanatomical features of HCM as a research field for disease-specific biomarkers; b) whether cardiac magnetic resonance (CMR) tissue characterization techniques hold potential to advance the discovery of circulating biomarkers, such as miRs, in HCM (Figure 1).

## Current biomarkers for HCM

Since myocardial biopsy is not typically used in managing HCM, biomarkers are sought from cardiac imaging, clinical features, and circulating molecules. Similar to other cardiovascular diseases, a multi-parametric approach is employed for the diagnosis and risk stratification in HCM.

The only validated diagnostic marker for HCM is LV myocardial thickness, defined as ≥15 mm in adult probands and routinely assessed using echocardiography or CMR [1]. However, this marker has limitations: it cannot always reliably distinguish HCM from secondary LV hypertrophy or metabolic disorders and is insufficient to detect preclinical and early-stage disease. Furthermore, the quality of echocardiographic image may be compromised by patient-related factors, while CMR, although more precise, is not universally accessible and has procedural restrictions. Supporting diagnostic tools include electrocardiogram, family screening, genetic testing, and clinical assessment to exclude phenocopies. Currently, there are no specific blood biomarkers for HCM. While several molecules reflecting myocardial wall stretch, fibrosis, inflammation, apoptosis, necrosis, and endothelial dysfunction have demonstrated correlations with imaging findings [15], they have not been integrated into clinical practice due to insufficient evidence of specificity, thereby limiting their diagnostic utility. Moreover, none of these biomarkers exhibit adequate sensitivity to detect subclinical HCM [16].

Few imaging markers and clinical features - such as ventricular tachycardia, unexplained syncope, and a family history of SCD - are incorporated into SCD risk stratification algorithms [17, 18]. However, these models do not include blood biomarkers and show modest predictive value. Current European guidelines recommend the use of N-terminal pro-B-type natriuretic peptide (NT-proBNP) and troponin T for assessing mortality risk in overt HCM, particularly for death due to heart failure [1]. Additionally, NT-proBNP can serve as a surrogate marker for evaluating the therapeutic efficacy of cardiac myosin inhibitors [19, 20]. However, no risk models are currently designed to predict heart failure or other non-SCD-related mortality in HCM.

Challenges in identifying blood biomarkers for HCM may be attributed to historically small sample sizes in cardiomyopathy studies, inaccurate phenotype characterization, and truly weak biomarker correlations. Furthermore, simplistic approach based on a limited set of pre-selected biomarkers may not capture the complex underlying pathways.

Recent advancements in omics technologies, enabling the quantification of thousands of low-abundance circulating molecules (e.g., RNA, proteins, metabolites), have led to the discovery of novel biomarkers. Studies have demonstrated the ability of compiled protein and miR panels to differentiate HCM from hypertensive heart disease [21, 22] and subclinical HCM from controls [23, 24].

## Circulating ncRNAs as biomarkers: case of miRs

Advances in cardiovascular genetics have shifted scientific interest toward the non-protein-coding genome and its non-coding RNA (ncRNA) transcripts [25]. The foremost function of ncRNAs is to regulate protein-coding gene expression, thereby affecting fundamental processes such as growth, differentiation, metabolism, apoptosis, and autophagy [26–29]. With growing evidence on the involvement of ncRNAs in heart diseases [30] and the advent of advanced computational methods, researchers can now explore the diagnostic, prognostic, and therapeutic potential of ncRNAs as biomarkers and novel targets for intervention [31].

Among various types of ncRNAs [32], miRs are the most abundant [33] and of particular interest in human diseases, including HCM [34]. MiRs are small molecules (∼20–22 nucleotides) that form a coordinated regulatory system and control gene expression post-transcriptionally by binding to their messenger RNAs, resulting in cleavage or translational repression [35]. A single miR can have numerous high- and low-affinity gene targets, and a single gene can be regulated by multiple miRs.

MiRs are detected not only in tissues but also in the bloodstream, making them attractive biomarkers for cardiac diseases, where biopsy is uncommon. They enter circulation from living and dead cells via active secretion or passive release. Despite the RNase-rich environment of blood, circulating miRs remain stable [36], protected within extracellular vesicles or bound to RNA-binding proteins [37, 38]. As conserved regulators of gene expression, miRs serve as dynamic biomarkers that reflect disease stages [39]. In heart failure studies, circulating miRs significantly improved the diagnostic power of NT-proBNP, which may be particularly beneficial for identifying heart failure with preserved ejection fraction, where standard clinical assessment, imaging, and NT-proBNP alone may not be definitive [40, 41].

### MiRs in HCM

Studies in animal and human tissues indicate that miRs significantly influence HCM [39, 42–48]. Several miRs, such as miR-1, -21, -30b, -132, -133a, -133b, -150, -199a-3p, and -486-3p, consistently show altered expression across at least two independent studies, suggesting a role in HCM development [39, 45, 47]. Plasma miR levels may reflect myocardial pathology, and around 30 circulating miRs have been identified as potential HCM biomarkers. However, the diagnostic accuracy of individual circulating miRs remains moderate [34].

Recent research has expanded miR panels to improve diagnostic accuracy. A six-miR set, including miR-181a-5p, -181c-5p, -328-3p, -301a-3p, -193b-3p, and -142-3p, outperformed individual miRs and differentiated sarcomeric variant carriers with and without the HCM phenotype with high statistical significance [23]. In a larger study involving 555 patients, transcriptomic profiling of 1,141 miRs identified a panel of 20+ circulating miRs that effectively discriminated HCM from hypertensive LV hypertrophy. Subsequent pathway analysis linked these miRs to key signaling pathways, including Ras-MAPK [22].

In our study, patients harbouring disease-causing *MYH7* variants had significantly higher plasma levels of miR-499a-5p compared to those with other sarcomeric variants, genotype-negative patients, and healthy controls [49]. MiR-499 is part of the “myo-miRs,” encoded by introns of cardiac myosin genes, including *MYH7*. These genes regulate muscle function by controlling the expression of both contractile proteins and regulatory miRs [50]. This finding supports a gene-oriented approach to studying miRs, as different genetic backgrounds may lead to distinct miR profiles that influence the disease phenotype.

### Limitations of circulating miRs for biomarkers

While circulating miRs hold promise as biomarkers, several challenges must be addressed before their clinical application. Unlike miRs measured in tissues, which can be cell-type-specific [51], circulating miRs often do not provide clear tissue- or disease-specific signals. Of the 2,600+ miRs identified, only a few - such as miR-1, -133a, -208a/b, and -499 - are categorized as cardiac-specific [52]. Inconsistencies in miR profiles between studies complicate their reliability as biomarkers. This variability may stem from differences in cohort characteristics, methodological processes, or the complex nature of miR regulation, where miRs typically have modest effects on many targets rather than having a dramatic impact on single genes. However, the combined effects of miRs on targets within a shared pathway can be synergistic [53], suggesting that miR panels, rather than individual miRs, could enhance diagnostic accuracy. Further challenges and solutions are detailed elsewhere [34, 54].

## Microanatomical features of HCM as a research field

Microanatomical changes in the myocardium, such as fibrosis, disarray, and MVD, are closely associated with HCM and its major clinical outcomes, as demonstrated in early histological studies [2–6], making them attractive substrates for the discovery of non-invasive biomarkers. Importantly, these changes are not merely secondary to LV hypertrophy but are also present in non-hypertrophied LV segments [55] and at the preclinical stage [56–58]. In mouse models, early administration of mavacamten, a first-in-class cardiac myosin inhibitor, suppressed the development of myocardial disarray and fibrosis by attenuating hypertrophic and profibrotic gene expression [59]. This highlights the role of specific signaling pathways in these adverse microanatomical changes in HCM. The identification of miR signatures associated with these features appears to be a promising avenue for HCM research.

### Non-invasive myocardial characterization with CMR imaging

CMR tissue characterization techniques offer a non-invasive, radiation-free assessment of fibrosis, microstructure, and microvascular health in HCM, with ongoing research improving their clinical utility.

Late gadolinium enhancement (LGE) is widely used to demonstrate replacement fibrosis, indicating scar tissue resulting from cardiomyocyte death. In contrast, interstitial fibrosis, which represents increased extracellular matrix and volume (ECV) without the pre-requisite cardiomyocyte death, is best evaluated using native T1-mapping and ECV measurements [60]. Both LGE (2-standard deviation technique) and T1/ECV-mapping have been histologically validated to accurately reflect myocardial fibrosis and collagen volume [6, 61–65]. Cardiac diffusion tensor imaging (cDTI) is an innovative CMR technique that assesses myocardial microstructure by mapping water diffusion along muscle fibers [66]. This method may reveal myocyte disarray, as demonstrated in preclinical models [67], although histological validation in humans is still needed. MVD can be assessed through perfusion CMR imaging, which quantifies myocardial blood flow, myocardial perfusion reserve, and perfusion defects [68]. It is important to adjust perfusion maps for LGE, as LGE contributes to resting perfusion defects in 30% of patients, potentially confounding the assessment of ischemic burden in HCM [69].

Although CMR-derived tissue parameters display specific features, they are not pathognomonic for HCM and can also be seen in other conditions. Caution is needed, as, for instance, markers like T1 relaxation time and ECV may reflect amyloidosis or edema. Tissue findings on CMR should be interpreted in the context of HCM and in conjunction with other markers, such as LV hypertrophy or the presence of sarcomere mutation.

The clinical application of CMR tissue markers in HCM is currently limited to fibrosis detection. The distribution and severity of focal and interstitial fibrosis aid in differentiating HCM from its phenocopies, such as Fabry disease and amyloidosis [70]. Extensive replacement fibrosis is increasingly recognized as a prognostic marker for SCD and all-cause mortality [71], with LGE >15% of LV mass now serving as a second-line indication for implantable cardioverter-defibrillators [17, 72]. T1 mapping assists in distinguishing HCM from hypertensive LV hypertrophy [73], while ECV has been associated with heart failure outcomes [74]. An ongoing large observational study (NCT01915615), integrating genetic, blood, and CMR markers - including LGE, T1 mapping, and ECV - is likely to offer further insight into their prognostic capabilities in HCM [75]. CMR-based assessment of MVD and myocardial disarray remains research-focused and is not yet part of the etiological diagnosis of HCM. However, to date, cDTI, although in need of technical improvements [66], has demonstrated the ability to discriminate preclinical HCM from healthy controls [67], and its correlation with ventricular arrhythmias highlights its prognostic potential alongside LGE and ECV [65].

## CMR tissue characterization techniques as a research tool in HCM

CMR, with its ability to accurately track pathological processes in the myocardium, is increasingly used in clinical trials of new therapeutics for non-ischemic cardiomyopathies [76]. CMR imaging series serve as surrogate markers of treatment efficacy and, as a merit, provide mechanistic insights into the molecular pathways of natural (placebo group) and treatment response over shorter time periods. In HCM, cardiac myosin inhibitors have been evaluated in clinical trials using surrogate clinical and imaging endpoints. The EXPLORER-HCM CMR substudy showed significant reductions in LV mass, wall thickness, and left atrial volume index, suggesting that mavacamten alters HCM pathophysiology [20]. Meanwhile, LGE and ECV were not significantly changed, supporting the irreversible nature of myocardial fibrosis. Recent research by Joy et al. showed associations of CMR-derived disarray and MVD with stages of phenotype evolution [67], suggesting potential future applications of these techniques in HCM research before and after therapeutic interventions. Notably, in overt disease, the presence versus absence of sarcomeric mutation has different effects on microstructure and microvasculature [67]. Stress perfusion CMR has recently been used as a validation tool for another potentially more cost-effective and clinically practical marker of MVD – impaired myocardial work on echocardiogram [77].

### CMR techniques for discovery of circulating biomarkers in HCM

Several conventional blood biomarkers have been shown in association with CMR-derived tissue characteristics, particularly LGE [15]. NT-proBNP and troponin T exhibit positive correlations with increasing LGE and ECV levels in a graded manner [78, 79]. Other biomarkers linked to necrosis (troponin I), fibrosis (matrix metalloproteinase-9, endostatin, apelin), inflammation and apoptosis (high sensitivity C-reactive protein, TNF-alpha), and endothelial dysfunction (big endothelin-1) show correlations with LGE and MVD [15], although validation in larger studies is required.

Proteomic and transcriptomic studies aimed at identifying biomarker signatures associated with adverse myocardial changes observed on CMR are a relatively new line of research. However, several studies have already been conducted in various conditions, including HCM.

A large study in healthy individuals identified a circulating protein signature associated with interstitial fibrosis. Prospective follow-up using progression to heart failure as an endpoint may provide validation for the discovered protein panel [80]. In patients with heart failure and preserved LV ejection fraction, unique biomarker patterns correlated with ECV (7 proteins) and myocardial perfusion reserve (6 proteins) [81]. In HCM, quantitative proteomics identified a six-biomarker panel related to myocardial substrate changes and SCD risk, with five of the six biomarkers elevated in subclinical HCM patients [24]. Proteomic profiling of 701 patients with sarcomeric HCM identified circulating biomarkers associated with adverse imaging and clinical phenotypes, including LGE [82].

A complementary CMR and transcriptome profiling approach identified a circulating miR signature as a biomarker for LGE-positive cardiomyopathy in muscular dystrophy [83]. In HCM, the relationship between miRs and tissue CMR parameters has been investigated in six studies, all of which had relatively small sample sizes and primarily focused on fibrosis, employing LGE and T1-mapping techniques [48, 84–88] (Table 1). Several candidate individual miRs with moderate to strong diagnostic value have been identified. Notably, the study by Fang et al. demonstrated the significant superiority of miR panels over single miR markers for diagnostic purposes. In that study, individual miRs showed moderate diagnostic performance for interstitial fibrosis (AUC 0.663–0.742), while combining eight miRs substantially improved the diagnostic accuracy (AUC 0.87) [84].

**TABLE 1:** Studies aimed at identifying MiR biomarkers associated with adverse myocardial changes observed on CMR in hypertrophic cardiomyopathy.

CMR tools	Studying myocardial characteristics	RNA class	Number of patients	Main conclusion	Ref
Postcontrast T1 mapping	Diffuse fibrosis if T1 time <470 ms	Circulating miRs	63 (+4 controls)	Individual miRs had moderate diagnostic value (AUC: 0.663–0.742), but combination of 8 miRs greatly improved diagnostic value (AUC 0.87) for the presence of diffuse fibrosis; 11 miR levels inversely correlated with T1 time	[84]
LGE	Focal fibrosis	Myocardial miRs	21 (+4 controls)	MiR-642a-3p expression was positively correlated to the quantification of LGE (r = 0.467)	[85]
LGE	Focal fibrosis	Circulating miRs	41 (+41 controls)	MiR-29a is significantly associated with both hypertrophy and fibrosis (r = 0.691)	[86]
LGE	Focal fibrosis	Circulating miRs	24 (+11 controls)	Elevated miR-4454 levels were significantly correlated with cardiac fibrosis (r = 0.560)	[87]
LGE	Focal fibrosis	Myocardial and circulating miRs	42 (+30 controls)	Circulating miR-221 is consistent with that in myocardial tissue, and correlated with myocardial fibrosis and hypertrophy (r = 0.630, AUC:0.764)	[48]
LGE	Focal fibrosis	Circulating miRs and lncRNAs	69	LncRNA-MIAT might be associated with the development of fibrosis in HCM via negatively regulating the expression of miR‐29a (AUC:0.810)	[88]

CMR, cardiac magnetic resonance; RNA, Ribonucleic acid; LGE, late gadolinium enhancement; miR, microRNA; lncRNA, long-noncoding RNA.

## Discussion

MiRs are promising biomarkers, as their altering profiles can reflect distinct molecular processes in the heart. Given the heterogeneity of HCM, likely due to a cascade of molecular and structural changes, it is essential to investigate biomarkers across all stages of the disease. Before the onset of overt LV hypertrophy, HCM is characterized by abnormalities in myocardial microstructure, which emphasize their relevance in early pathogenesis. We presume that circulating molecules associated with these specific changes could be the strong candidates for further validation in larger studies as both disease-specific and prognostic biomarkers.

Given the limitations and biases of myocardial biopsy, CMR serves as a valuable tool for cardiac research. Advanced CMR technologies, currently being validated in preclinical models and human histology, enable the non-invasive visualization and quantification of myocardial microanatomical changes at all stages of the disease, including the preclinical phase. Preclinical HCM appears to be of particular interest for biomarker discovery, offering a unique opportunity to explore ncRNA signatures and potentially uncover disease-specific pathways without the confounding influence of secondary changes related to hemodynamic abnormalities. The expanding availability of genetic testing, including cascade family screening, is facilitating the identification of mutation carriers, making such studies feasible. When conducting discovery studies in overt HCM, the genotype of patients should be considered, as both miRs and myocardial microstructure are sensitive to the genotype status.

Novel HCM therapeutics, such as myosin inhibitors and gene editing, hold the potential to reverse the disease phenotype. Incorporating candidate miR panels into such self-controlled trials could enhance biomarker discovery by identifying those that reflect reversible adverse myocardial changes. A complementary imaging and genome-based biomarker approach could deeper insights into the complex underlying processes and identify novel targets for emerging therapeutic technologies.

HCM is a slowly progressive disease with a low event rate, making prospective studies is costly and time-consuming. Nevertheless, longitudinal studies with adequate sample size are essential to evaluate the prognostic power of candidate miR panels. Although conducting such studies in HCM is challenging, growing awareness and diagnostic expertise among professionals, emerging international cardiomyopathy collaborations, and advances in potentially curative therapies foster optimism.

Besides miRs, there are two other types of ncRNAs in the scope of interest in HCM: long non-coding RNAs (lncRNAs), which account for 80%–90% of all ncRNAs, and circular RNAs (circRNAs), a newer class of ncRNAs known for their stability due to a closed ring structure [89, 90]. To the best of our knowledge, only one study has investigated myocardial fibrosis in HCM by integrating CMR with relevant circulating lncRNAs [88]. This field remains largely unexplored, and significant efforts are required to advance our understanding.

## Conclusion

Enhanced myocardial characterization and staging of HCM using advanced CMR techniques holds promise for identifying circulating miRs as biomarkers. MiR signatures associated with adverse microanatomical changes detected by CMR could be the strong candidates for longitudinal validation studies. To ensure comprehensive and reliable results, future research should consider patients’ genetic status.
